# Mouse Activity across Time Scales: Fractal Scenarios

**DOI:** 10.1371/journal.pone.0105092

**Published:** 2014-10-02

**Authors:** G. Z. dos Santos Lima, B. Lobão-Soares, G. C. do Nascimento, Arthur S. C. França, L. Muratori, S. Ribeiro, G. Corso

**Affiliations:** 1 Escola de Ciências e Tecnologia, Universidade Federal do Rio Grande do Norte, Natal, Rio Grande do Norte, Brazil; 2 Centro de Biofísica, Universidade Federal do Rio Grande do Norte, Natal, Rio Grande do Norte, Brazil; 3 Departamento de Engenharia Biomédica, Universidade Federal do Rio Grande do Norte, Natal, Rio Grande do Norte, Brazil; 4 Brain Institute, Universidade Federal do Rio Grande do Norte, Natal, Rio Grande do Norte, Brazil; 5 Center for Polymer Studies and Department of Physics, Boston University, Boston, Massachusetts, United States of America; University of Alberta, Canada

## Abstract

In this work we devise a classification of mouse activity patterns based on accelerometer data using Detrended Fluctuation Analysis. We use two characteristic mouse behavioural states as benchmarks in this study: waking in free activity and slow-wave sleep (SWS). In both situations we find roughly the same pattern: for short time intervals we observe high correlation in activity - a typical 1/f complex pattern - while for large time intervals there is anti-correlation. High correlation of short intervals (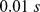 to 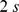: waking state and 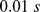 to 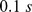: SWS) is related to highly coordinated muscle activity. In the waking state we associate high correlation both to muscle activity and to mouse stereotyped movements (grooming, waking, etc.). On the other side, the observed anti-correlation over large time scales (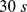 to 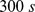: waking state and 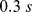 to 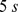: SWS) during SWS appears related to a feedback autonomic response. The transition from correlated regime at short scales to an anti-correlated regime at large scales during SWS is given by the respiratory cycle interval, while during the waking state this transition occurs at the time scale corresponding to the duration of the stereotyped mouse movements. Furthermore, we find that the waking state is characterized by longer time scales than SWS and by a softer transition from correlation to anti-correlation. Moreover, this soft transition in the waking state encompass a behavioural time scale window that gives rise to a multifractal pattern. We believe that the observed multifractality in mouse activity is formed by the integration of several stereotyped movements each one with a characteristic time correlation. Finally, we compare scaling properties of body acceleration fluctuation time series during sleep and wake periods for healthy mice. Interestingly, differences between sleep and wake in the scaling exponents are comparable to previous works regarding human heartbeat. Complementarily, the nature of these sleep-wake dynamics could lead to a better understanding of neuroautonomic regulation mechanisms.

## Introduction

In the last two decades the influx of ideas from complex systems physics have brought new insights into biomedical studies [1]–[5]. The concepts of fractals and structure of fluctuations have enlightened analysis of physiological time series, for instance: heartbeat series [6]–[8], breathing records [9]–[11] and gait dynamics [12]–[15]. These three physiologic records have in common the absence of typical periodicity that could guide the characterization of the dynamics, and because of that, scientists are driven to the study of signal fluctuation. Signal fluctuation is a mathematical technique that classify fractal patterns of time series [16]–[19]. The output of this methodology is a fractal analysis, the extreme case of absence of order, or pattern, in signal is the white noise which has a well-defined dimension. Most intricate scenarios can rise like different fractal dimensions for distinct time intervals or multifractality - a set of fractal dimensions [1], [20]. Beyond fractal dimensions, this paper is centred in exploring time scales of distinct fractal regimes and, especially, transition times between fractal scenarios considering typical states of mice sleep-wake cycle.

Specifically, in this study we focus on scaling and multifractal features in locomotor dynamics of mice activity. This approach can be useful to understand the neural, as well as environmental, aspects related to body movement in different physiological conditions. We lead in this work with accelerometer time series recorded with a device placed on the head of a mouse. Previous similar studies analysed nonlinear (multifractal) properties of heartbeat physiological dynamics [1], [6], as well as monofractal 1/f type scaling in the gait of adult individuals [14], [15]. Furthermore, multifractal behaviour was found in children gait and were gradually lost with maturation [14]. There are also earlier studies on wrist and arm motion, which, in contrast to adult gait, reported nonlinear scaling [21]. Acceleration is a measure of animal activity, and in this way our work interfaces with the cited works about heart, breath and gait dynamics. We employ the raw accelerometer data instead of computing active versus inactive intervals as in references [22]–[24] because we intended to capture detailed activity patterns of individuals. In addition, our study encompasses nine orders of magnitude in the time record, from 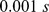 to several hours. In this way our analysis capture activity bellow the time between two successive heartbeats to the entire duration of sleep-wake cycle state. The novelty of our work consist in using fractal concepts to analyse motor activity by taking into account relevant physiologic durations like: the inter heartbeat interval, the breathing period, the typical time of stereotyped animal movements and the wake sleep duration cycle.

The main concept we employ in the analysis the accelerometer data is the fractal dimension which is connected to signal fractal correlation [16]. The human gait is a typical expression of motor system phenomenon that exhibits high correlation [14], [25] which reflects the repetition and coordination of the integrated action of neural and muscular system. The most illustrative picture of a high correlation movement comes from a young person walking for a short period (around 1 hour) without resting [12], [14]. For an older person the gait looses correlation; the image of a limping old man whose next step seems not follow the previous one is a powerful picture of absence of correlation. Human gait also produces a somewhat artificial, but illustrative, example of anti-correlation which comes from subjects that should, by suggestion, follow an arbitrary synchronized signal; in this case, the individual attempt to correct himself to adjust the step producing a negative feedback which induces an anti-correlation trend [12]. The examples of correlation and anti-correlation we have found in the free range behaviour mouse are similar to those of human gait. The correlation may be generally associated to skeletal activity patterns usually present in individual body movement. Complementarily, the anti-correlation may arise as a consequence of a negative feedback normally associated to the fatigue of movement, heartbeat or breathing.

In the analysis of signal auto-correlation we are driven to the concept of fractals; in this context, correlated, no-correlated or anti-correlated signals are characterized by power-law and distinct fractal dimensions [20]. Indeed, there is a plethora of different possibilities of autocorrelation signal characteristics. As an illustration, a monofractal signal identifies an unique self-similarity index, which can correspond to correlated, no-correlated or anti-correlated behaviour. A bi-fractal data discloses two sub-sets with different correlations and fractal dimension according to the time scale and these different behaviours are typically separated by a crossover time. A multifractal time series comprises a set of many fractal dimensions for the same time scale [26].

Extensive analysis has shown that different correlation exponents in DFA scaling characterize the heart rate of healthy subjects and patients suffering from heart disease. Also, different exponents have been found for the wake and the sleep periods. Moreover, the crossover phenomenon in DFA scaling was reported in real physiological signal when comparing scaling properties of the cardiac dynamics during sleep and wake periods [27], [28]. Furthermore, crossover patterns were observed in changing regimens from rest to exercise (heartbeat fluctuations) [29], between different sleep stages [11], [30] and even across circadian phases of cardiac (eletroencephalographic signal), breathing (respiratory parameters) and locomotor dynamics [31]. Besides, we are aware that the crossover might be an artefact of oscillatory patterns embedded in the signal. The crossover artefact was theoretically investigated [17], and also has been shown for real heartbeat data [25]. On the other hand, scaling crossovers in general locomotion may be related to intrinsic mechanisms of physiological regulation, such as suprachiasmatic nucleus circadian regulation and other neural output variances [32]. Thus, we hypothesise that mouse locomotion dynamics during waking may disclose a complex temporal organization characterized by a scale-invariant fluctuation across a range of time scales.

In this paper we work on a comprehensive analysis of motor activity of mice in free range environment using fractal and correlation concepts. We propose that signal correlation analysis could provide new insights not only for motor activity studies but also into behavioural description of rodents. In addition, fluctuation analysis can be used to identify and quantify patterns of accelerometer records, in other words, a standard model of motor activity based on time scales. Finally, the accelerometer-based DFA analysis can be used as a robust tool for evaluating promising therapies in models of motor alterations induced by drugs or in neuromuscular diseases. Some rodent models of demyelinating pathologies or neurodegenerative disorders such as Parkinson disease could be good examples of a possible benefit on this technique in a near future.

## Results

**Figure 1:**
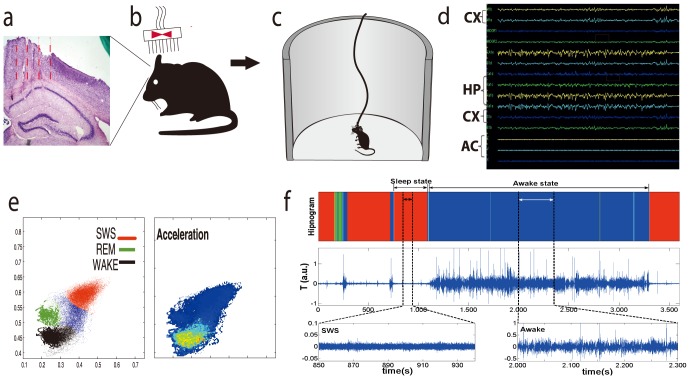
Sequence of animal procedures and data acquisition. (a) Histological analysis. After all behavioural procedures and data collection, all implanted C57-BL6 mice were perfused and brain slices were obtained in order to confirm if LFP electrodes were inserted in M1 and S1 cortical regions and in hippocampus CA1 subfield as an inclusion criterion. (b) Surgical Matrix Electrode Implant: Under a isofurane anaesthesia, mice a rectangle was opened in cranial bones for allowing a 16-tungsten electrode matrix implant combined with an accelerometer in the headstage. Eight electrodes were placed in the S1/M1cortex (layers 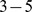) and another eight in the CA1 subfield of hippocampus. (c) Electrophysiology. One week after surgery, animals were submitted to a session of 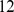 hours continuous recording in a round open field maze. They were allowed to perform their natural behaviours during the recordings, and to freely display the sleep-wake cycle. (d) LFP oscillations. All channels of hippocampus, cortex and accelerometer were displayed in real time analysis in order to verify possible problems with ground or signal generation. (e) State map generation. Real-time two-dimensional behavioural state maps were generated by plotting the following spectral ratios: x-axis, 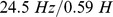
z; y-axis, 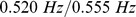. Raw LFP and EMG activity were analysed during periods of WK, SWS, and REM sleep predicted by the two-dimensional state map. (f) A typical AR (midlle) with the hypnogram generated by identification of waking (blue), SWS (red) and REM (green) sleep cluster separation (top). At bottom two zooms of the raw accelerometer signal for resting SWS state (left) and waking state (right).

**Figure 2:**
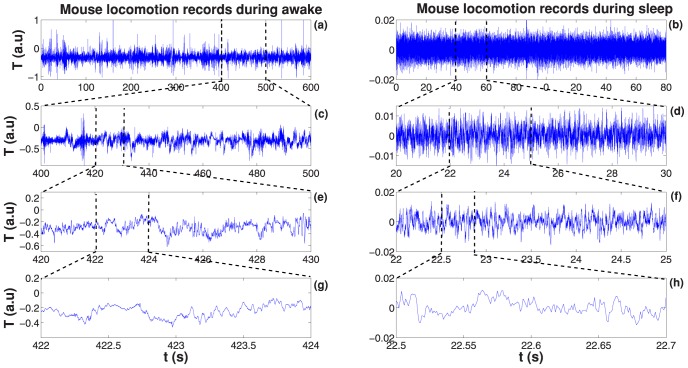
Accelerometer time series. (a) Sample of a time interval (600 seconds) record during a typical wake fluctuation activity for a healthy mouse. In (c), (e) and (g) we show segments of the time series for the same wake state period at small time scales to visualize self-similar (fractal) fluctuation. (b) Representative of a time interval (90 seconds) record during SWS sleep stage for the same mouse. In (d), (f) and (h) shows a sequence of segments in many time scales of this fluctuation. These fluctuations are typical examples of noise-like time series.

A typical time series of accelerometer record encompassing one hour is shown in Fig. 1(f). To facilitate the comparison between these data and behavioural states, hipnogram is depicted in the same plot. A visual analysis points out, as expected, that the intensity of accelerometer response is higher when the individual is awake as compared to SWS state. Our analysis was done independently for resting SWS and waking states, otherwise it is not possible to find defined patterns in the results. The two case situations are: the individual in waking state without any sleep episodes [see Fig. 2(a)] and in resting sleep state [see Fig. 2(b)], that means, the sleeping individual that does not exhibit any macroscopic movement (no arousals). We have identified these two states as benchmarks we can use to classify the phenomenology of actimetry (accelerometer records). In the following we will always refer to these states as the waking and resting SWS states, since each of this states show common characteristics. It is important to highlight the universality of our results: all time series we have analysed for all individuals share the same basic phenomenology in the waking and resting SWS states.

**Figure 3:**
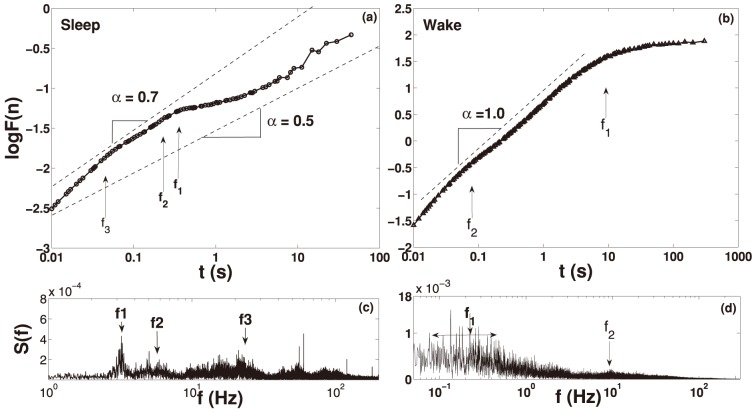
DFA analysis and Power Spectrum. In (a) we show the DFA fluctuation function 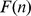 of locomotor fluctuation during sleep for the time series presented in Fig. 2(b). In this figure we present the x-axis as a function of time in seconds, instead of points (1 second corresponds to n = 1000 points). In (c) shows the Fourier spectrum that display three characteristic frequencies highlighted by arrows (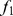, 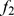 and 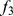). These frequencies are the most probable cause of the 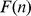 hump. The main frequency seems to be related to crossover at approximately 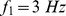 which is the breathing cycle. Other two frequencies may match the oscillation pattern of the heartbeat (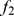) and physiological tremor (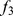). We also note that for the full time scale the coefficient slope is 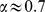. In (b) we show the DFA of locomotor fluctuation during awake from time series presented in Fig. 2(a). In (d) shows the Fourier spectrum that also display two different frequencies which are highlighted by arrows (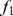 and 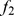). The 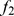 frequency, typical of the heartbeat, causes a slightly 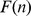 hump at approximately 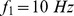. The set at 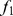 indicates a range of frequencies from 0.05 Hz to 0.7 Hz that match the oscillation pattern of physiological activities and stereotyped movements. We also note that for time scale up to 7 seconds the coefficient slope is 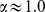.

The essence of results concerning signal fluctuation structure is shown in Fig. 3. In this figure we explore Fourier and DFA results of a typical waking (b)–(d) and resting sleep signal (a)–(c); the signals themselves are shown in Fig. 2(a)–(b). We should invert the time reading of the x-axis when we go from DFA to Fourier pictures, for instance, a time 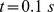 gives frequency 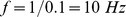. The values of the DFA exponent, 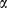, corresponding to the slope of the DFA curve are shown in Fig. 2(a)–(b) (dashed lines - as an eye guide). Note within the DFA graphic as well the Fourier spectrum three different frequencies highlighted by arrows (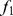, 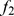 and 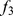). These frequencies may be related to DFA humps and the crossover at approximately 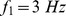 due to an oscillation pattern given by breathing [15]. The other frequencies match the oscillation pattern of the heartbeat 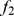 and muscle tremor 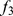. We also see that for all time scale the mean coefficient slope is 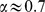. In 3(b) we show the DFA and Fourier spectrum of locomotor fluctuation during awake. We indicate in this figure two frequencies (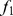 and 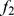). The 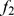 frequency may cause a small DFA hump at approximately 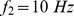 and it is associated to the heartbeat oscillation. The another 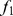 is actually a range of frequencies from 0.05 Hz to 0.7 Hz that match the oscillation pattern of physiological activities and stereotyped movements. We also note that for time scale up to 7 seconds the coefficient slope is 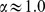.

**Figure 4:**
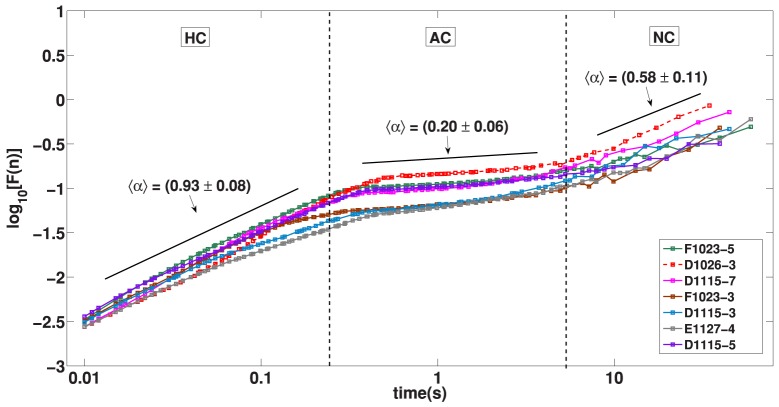
DFA: Resting SWS State. The fluctuation function 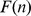 versus the time scale size 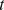 (1 second corresponds to n = 1000 points) in double logarithmic scale. The dotted lines shows the regions with different regimes. HC indicates a range in time-scale (from 0.01 s to 0.3 s) with highly correlated regime corresponding to 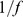 noise (fractal range). AC indicates a range in time-scale (from 0.4 s to 5 s) with anti-correlated regime. NC indicates a range in time-scale (above 10 s) with descorrelation regime.

**Figure 5:**
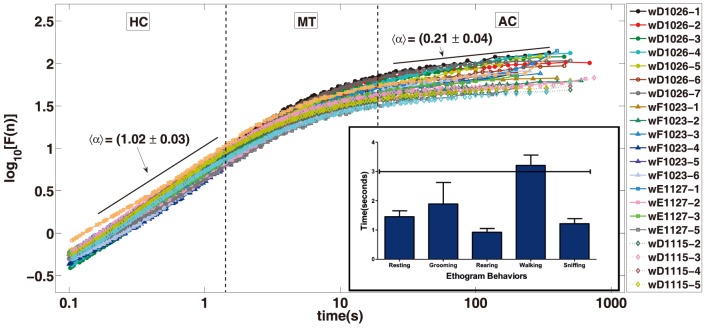
DFA: Waking State. The fluctuation function 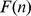 versus the time scale size 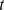 (1 second corresponds n = 1000 points) in double logarithmic scale. The dotted lines shows the regions with different regimes. HC indicates a range in time-scale (from 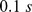 to 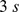) with highly correlated regime corresponding to 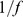 noise (fractal range). MT indicates a range in time-scale (from 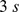 to 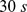) with multifractal behaviour. AC indicates a range in time-scale (from 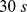 to 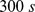) with anti-correlated regime. Furthermore MT is a long transition regime from HC to AC regimes. In the low position at right side are inserted the main stereotyped behaviours with their average duration and standard error medium. Please note that their duration in general are less than 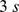, which fits on HC regime. The inset shows a histogram of duration of mice stereotyped movements.

**Figure 6:**
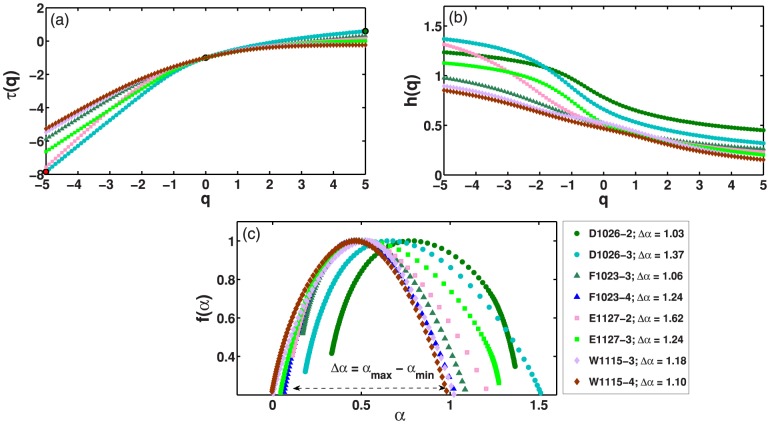
Multifractal detrended fluctuation analysis (MF-DFA). (a) Generalized Hurst exponent h(q) for eight selected data series corresponding to 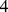 mice. (b) Similar plot for the multifractal scaling exponent 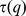. (c) Multifractal spectrum 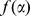 for the same signals. Here, h(q), 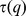, and 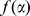 are obtained, respectively, through Eqs. (7), (8), and (10), and 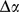 corresponds to the width of the multifractal spectrum.

The analysis of fluctuations of the accelerometer signal reveals the same basic structure for waking and SWS states. In both situations we found the presence of an Highly Correlated (HC) signal at low time scales while intermediate time scales reveals an Anti-Correlated (AC) patterns and very long times are marked by a Non Correlated (NC) signal. For the waking state the passage from HC to AC is not abrupt, we identify here a Multifractal Transition (MT) which reveals a great richness of distinct correlations. We remark that, despite a similar HC, AC and NC pattern, the waking and sleep states differ regarding their time scales and phenomenology. The time intervals for sleep states are typically shorter than waking states what is expected since sleep is mainly regulated by autonomic activity which are dominated by smaller time scales as compared to behavioural times. In what follows we show in detail these patterns that are illustrated in Fig. 4 and Fig. 5.

(HC): The DFA technique for short times reveals a very high correlation response of the accelerometer signal. The HC is found from 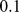 to 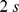 in the waking state, Fig. 5, and 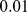 to 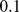 in sleep state, Fig. 4. This result follows the strongly correlated contraction pattern of the muscle skeletal activity. For small time scales the muscle skeletal activity is very coordinated, the short time scale of motor activity can be imagined as a chain of small movements that follow each other by necessity; for instance, each animal limb movement is strongly correlated to the movement of the limb in a previous instant. This simple picture of animal motor activity points to a high correlation in the sequence of intervals of movement. Of course, for long times individuals will follow another movement pattern breaking the correlation. In the sleep state the movement is dominated by heart and breathing movements as well as thermogenesis processes (tremor, vibration etc) which also implies in strong coordinate activity.

(AC): The high correlation of short time scale follows an anti-correlated regime. For the sleep state the transition from HC to AC can be characterized by a crossover while in the waking state the transition is smooth; a detailed approach of this transition reveals a rich multifractal structure which we discuss in detail later. The time scale of the AC regime is quite different in the two situations, for the waking state it goes from 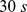 to 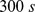 while in the sleep case it ranges from 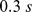 to 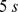. The physiology behind the AC show some similarity in waking and SWS states. For the waking state the AC regime is believed to be related to a cycle of exercise and rest, once the motor physiology of free range animals normally shows this oscillatory pattern. The apparent alternation between periods of intense and relaxed activity produces a natural anti-correlated patterns in the signal. For the SWS state the anti-correlation is similar but it is probably related to the autonomic system and it is rooted in the sympathetic parasympathetic regulation of cardiac and breathing system. In a similar way, an intense cardiac activity should be succeeded, via feedback control, by a diminution in the heart rate and vice-versa [7].

(NC): Considering long enough time scales the accelerometer signal should loose completely its correlation. In other words, for long times the memory of initial instants should vanish. The paradigmatic signal that shows this behaviour is the white noise whose signal at time 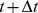 is completely independent of signal at time t, for 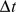 the time step. The absence of correlation in the waking regime comes after a 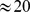 minutes while in the sleep state it happens after 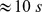. The presence of NC in the experiment is important for checking the consistency of our results.

(MT): The multifractal transition is present only in the waking regime. This regime is characterized by a set of different correlated patterns superposed in a same time scales that ranges from 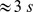 to 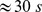. In our observations most of the stereotyped behaviours of mice such as scratching, roaming, short walking, sniffing, span below 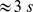. Inside each of these stereotyped behaviours the animal activity is in the HC regime, but the composition of several stereotyped behaviours show a more involved mathematical description. Indeed, we have found that in the typical behavioural range 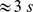 to 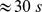 where more complex phenomena take place, the animal should aggregate several stereotyped behaviours. In a behavioural perspective the succession of stereotyped movements of the MT is related to the voluntary movement of individuals. The result of this composition, each one with its proper correlation length and fractal fluctuation signature forms the multifractal pattern shown in Fig. 6.

Fig. 5 shows the DFA picture for 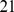 signal samples of the waking state corresponding to 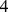 individuals. It is notable the qualitative agreement among the curves; the HC, MT, AC sequence is clearly visible in all samples. The DFA parameter for the HC interval is 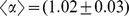 while for AC is 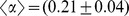. With an auxiliary line we plot the slope, computed as the average of slopes, for HC and AC regimes. We notice that the passage from HC to AC is a curve with a smooth slope variation. In the following we mathematically describe in detail this time interval that extend approximately for 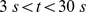. The absence of a defined slope in this interval unveils a richer geometric structure that deserves to be understood in a physiologic context. In the next paragraph we explore the computational techniques showing that, in this interval, the signal is multifractal. Indeed, the HC and the AC regimes are monofractal signal, that means, it is possible to full characterize the signal with a single index the fractal dimension of the signal which is not the case of the multifractal regime.

Multifractal behaviour in the dynamics of mice activities can be also verified through the generalized Hurst exponent 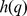, multifractal scaling exponent 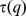, and multifractal spectrum 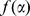, respectively obtained through Eqs. (7), (8), and (10). Fig. 6 shows 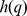, 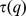, and 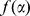 for time series of mice activities obtained of time interval that extend approximately for 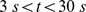. In this case, it is interesting to note that the decrease of 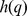 with an increasing 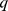 and the non linear behaviour of 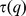 with 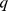 are observed for all mice activity samples. The relation between 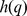 and 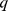 leads to a linear dependence of 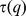 with 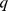. Moreover, the linear dependence gives rise to a multifractal spectrum 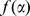 given by a small arc, with small width 6. The absolute value for the width of the multifractal spectrum 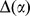, that corresponds to the difference between the maximum and minimum 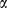, as well as the shape of the multifractal spectrum, are related to the temporal variation of the generalized Hurst exponent 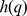 [18]. Furthermore, the degree of multifractality is given by the calculus of the absolute value of the spectrum width. In this sense, the wider 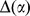, more significant is the multifractality of the analysed time series [33]. Here, the obtained width 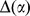 above 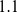, as signature of a rich and strong multifractal behaviour, irrespective of the individual.

Fig. 4 show the DFA graphic for seven signal samples of the resting sleep state. In this analysis we have used samples of four individuals. The curves show a typical HC, AC, NC sequence. Diversely from the previous regime, the transition from HC to AC shows a crossing over pattern in contrast with the smooth transition of the waking state. The resting sleep state can be characterized by three monofractal regimes in opposition to the waking state that needs a multifractal regime in the HC to AC transition. The 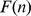 that represent fluctuation function is diverse among the states. In the top of the figure, with the highest fluctuation there is the waking state, a similar picture is shown in Fig. 5. At the bottom of the same figure we have the resting SWS state with lowest fluctuations, these two curves are similar to the ones shown in Fig. 4. Above the resting SWS state we have two curves corresponding to individuals that sleep but present measurable movement. The fluctuation caused by small movement of limbs hide the AC pattern in this situation. For this state the correlation, caused by the episodic movement in the sleep, prolong the correlation in the signal for larger times. Above the SWS state and bellow the waking state we have two curves corresponding to individuals that mix waking and sleep patterns. Obviously, because of the higher fluctuation of the waking state signal dominates the statistics the DFA resemble closely the one of the waking state, but 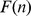 is smaller. We note that for long times individuals will show episodes of sleep and waking states and, by consequence, the accelerometer signal will necessarily loose all correlation; this situation is indicated in figure by the line 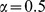.

## Discussion

The accelerometer analysis can be an useful, low cost and non-invasive technique that provides important hints about motor state and behaviour of a species. The main objective of this paper is, using fluctuation analysis, to propose a basic description of accelerometer measurements for a freely behaving mouse. As far as we know, this is the first time that DFA is being used to describe mice physiologic motor patterns using accelerometer signal data, an interesting way to classify activity patterns using neural networks is done in [34]. We design a classification of activity records using two benchmarks: the free waking and the SWS resting state. However, in both sleep or waking states, we observe the same tendency: small time lags are dominated by strong motor correlation while larger times are marked by anti-correlation. For large enough periods (above 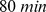), signal becomes uncorrelated as it should be expected for this methodology. The long term objective of this study is to provide a setup to help scientist to use accelerometer records as a safe tool to understand the effect of drugs or physical therapeutics in the motor neural system. Our group, in special, intends to study drugs that simulate Parkinson disease which is well known to have a profound impact on organism motility. In the following lines, we discuss fluctuation correlation regimes in the light of physiological and behavioural events starting from small to larger time scales, and considering separately waking and sleep states.

### Waking: Physiological activity and stereotyped movements

**Figure 7:**
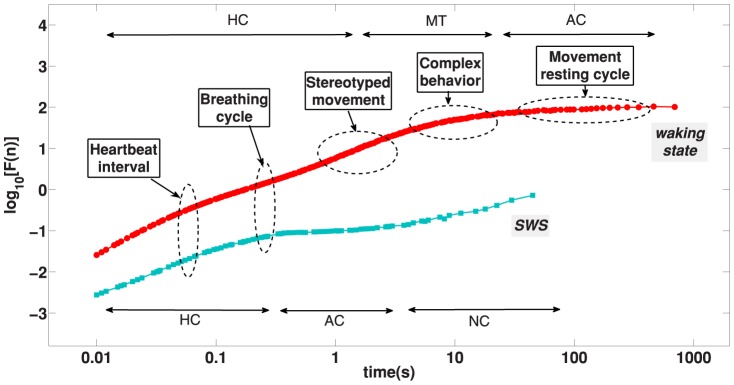
DFA activity analysis related to main physiological and behavioural events. In this picture we put the main physiologic and behavioural durations associated with the DFA accelerometer data curves. On the upper position we plotted regions of HC, MT and AC for waking state curve (shown in red), on the lowered position are indicated the HC, AC and NC regimes for resting sleep curve (in light green). For the resting SWS state the main physiologic event is the breathing cycle while for the waking state the main event is the behavioural time.

When we look at the waking DFA curve (Fig. 5) the first scale from 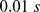 to 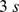 is an HC like regime. We situate in this interval two important physiological events: the heartbeat and breathe movements. Because in both waking and SWS recordings, heartbeat and breathe movements are set on the first HC part of DFA curve, we discuss in more details their influence when we reach SWS analysis. Another important transition point is related to mice stereotyped behaviour. Typical examples of stereotyped behaviour for this species are: short walks, grooming, rearing, resting and sniffing. We measure the duration of the stereotyped behaviours in our experiment in the interval ranging from 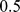 to 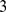 seconds, see Fig. 5 and 7. The average duration of stereotyped movement (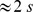) defines also a turning point in the DFA of waking state (Fig. 5). Indeed, until the stereotyped movement duration waking activity is HC; in this regime the recorded activity is strongly related to skeletal muscle coordinated movement of body and limbs. We state that, in a time scale below the average duration of stereotyped behaviours, the DFA pattern is roughly monofractal, while after this 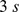 breaking point (an interval from 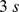 to 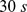) we see in the curve an evolution to a more complex pattern. According to this view, in waking state, the accelerometer amplitude of these behaviours are so high that surpass the input that result from cardiac or respiratory movements. Stereotyped and basic movements usually depends largely on automatic control programs situated on basal ganglia and lower parts of the brain [35]. Based on environmental output, internal valuation of actions and action selection, the animal may decide what will be the next movement pattern, which can be simple, frequent and stereotyped, or it can be less frequent and complex. The observation of Fig. 5 (inset) allows identification of a general duration of commonly observed mouse ethogram parameters.

### Waking: Complex behaviours

For the waking state we identify a distinct regime that start in 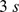 and goes to 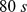 that we call here complex behaviour window. This regime has a proper physiological interpretation as a composition of several stereotyped movements and gives origin to a multifractal scenario. Let us consider a rough image of fluctuation analysis technique to understand this complex behaviour. The DFA analysis is about correlation statistics over different time scales, beginning with small, milliseconds intervals, and finishing with durations of many hours. For the waking state a duration of 1 s in the accelerometer recording will probably be related to data inside a given stereotyped behaviours. On the other side, a time window of, for instance, 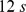 should fulfil a sequence of four or five diverse stereotyped movements; a statistic over these different stereotyped behaviours will not produce an unique fractal exponent, but reflect the variability of this bundle of stereotyped movements, each one with a characteristic time correlation [36]. For instance, given the stereotyped behaviours A, B, C, D, possible sequences reflecting the observed variability in a 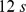 interval could be A, C, D, A; C, A, D, B; or D, A, D, C and so on, thus increasing variability and unpredictability in DFA curve. Of course, we cannot state that complex behaviour is composed exclusively by sequences of stereotyped behaviours. Complex behaviours may emerge as a natural tendency of experimentation and are associated to the learning of the environment by inspection and are associated with exploration of novelty. In this study case, novelty was linked by the round maze, which was an unknown environment for the mouse in the first minutes of exploration. For avoiding the influence of a movement pattern linked to novelty exploration, we removed the first hour of mouse activity, in which the novelty-driven exploration behaviour is more relevant. This procedure allowed us to focus on a movement pattern that is evoked when the animal is more habituated with the environment, and thus could better reflect the movement pattern which occurs more frequently in the animal routine environment.

### Waking: Movement and Resting (energy saving balance)

The accelerometer record reveals an interesting physiologic feature for durations from 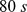 to 1 hour: the activity is anti-correlated, AC. The first AC record in physiology using fluctuation analysis was noticed in long range human heartbeat data [7]. In the cited work the author claims the autonomic system is responsible for the autocorrelation regulation. In this work we believe that a similar phenomenon is rooted in a general energy saving balance principle common to life organisms. In studies of locomotion related to free exercise in a running wheel mice and other rodents exhibit a voluntary drive for exercise, which are performed in temporal cycles or running and rest [37], [38]. Complementarily, studies concerning locomotion in other species highlight that animals present different moving patterns in their environments. This is probably because they consider the amount of energy they could intake during food search and the exploratory risk which vary along time [39]. We believe that these biological factors could be involved in the anticorrelation we found in waking, starting from the animal drive for exploration of a new environment allied with the demand for exercise, until the habituation with the environment, and a need for rest after a period of exploration. However, the presence of an AC regime in accelerometer record was not observed in human studies [12], [13] probably because most records consists of individuals walking without resting evaluation. We believe that future experiments designed to observe rest and movement cycle in free behaving humans and other species should also find an AC pattern as we are now describing for mice.

### Resting SWS

Starting with small periods, as occurs in waking state, the first relevant physiological parameter is the heart beat. We situate mice heart rate in a 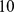 to 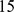
Hz interval (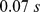 to 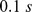) [40]. Basal heart rate is essentially dictated by atrioventricular and sinoatrial peacemaker activity [41], [42], which occurs by spontaneous activity of autorritmic cells and nervous system autonomic control [42], [43]. The observation of Fig. 4 shows that the effect of the heart beat seems not modify the slope of the DFA curve both to SWS and waking state. This might be due to the fable movement amplitude of this signal considering that the accelerometer is situated on individuals head. The major crossover time of fluctuation analysis of the resting SWS state is given by the breathing duration cycle, that immediately follows heartbeat interval. Above breathing period we capture the oscillatory muscle activity of several breathing cycles; bellow this duration the accelerometer records the muscle movement inside each breathing cycle. The movement inside breathing cycle is a typical HC patterns that is present in any highly coordinate skeletal muscle activity. The respiratory movement has a frequency from 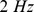 to 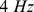 (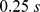 to 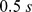) [42] in mice. We believe that, due to a larger movement amplitude driven by breathe muscles, the effect of respiratory weight in accelerometer records is more intense than cardiac stimuli.

We discussed above that AC regime in SWS state can be interpreted as an artefact caused by the periodicity of breathing cycle. However, we also point out the possibility of AC phenomenon be a true feedback mechanism. Respiratory rate is controlled by specialized neuron nuclei localized in the pons and medulla oblongatta, such as pre-Böltzinger and Böltzinger complexes. These nuclei are probably responsible for different rhythm patterns, and receives also influences from other parts of the brain. These connexions can include chemoreceptors of retrotrapezoid nucleus, which are sensitive to 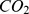, and Ph variation. In this way, variation of blood gases are linked to an automatic response feedback mechanism which regulates respiratory rate. Neurons coming from superior centres such as cortex and cerebellum also modulate basic respiration rhythms [44]–[46] and this input are also related to muscle effort and complex movements [47]. In special, since the perception and response to oxygen and carbonic acid blood concentration may occur starting from a half second (the time space of a breathe complete cycle), this mechanism may lead per se to an alternation of breathing frequencies in a SWS or waking resting state. The variation of inputs on respiratory nuclei associated to the chemosensory feedback response might explain our findings of anti-correlation during SWS sleep.

In addition, the most prominent peaks observed in SWS Fourier analysis (Fig. 3), could be associated to breathe (first), heartbeat (second), and to physiological tremor frequencies (third). In this context, it is important to highlight that physiological tremor in mice, which has origin on basal muscle frequencies [48], [49] can represent an important source of variation of the raw signal. This physiological frequencies also vary according to the muscle type [50], [51]. We emphasize that this tremor can be perceived simply by placing an habituated mouse on the hands of the researcher.

Finally we compare the scaling properties of the activity fluctuation during sleep and wakefulness time intervals in healthy mice. Remarkably, the difference in scaling behaviour between sleep and wake periods is comparable to previous works on the human heartbeat [27], [28]. Our outcomes corroborate these works that show the difference between the coefficients around 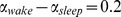. Our results shows a difference around 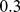 [see Fig. 3]. Elucidating the nature of the sleep-wake dynamics can lead to a better understanding of the mechanisms of egulation of autonomic neuro-motor activity. Furthermore, these results indicate that in the transition from sleep to wake the observed phase transition in the scaling behaviour across time scales from lower to higher scaling exponent does not depend on species characteristics (whether human or mouse) that are associated with fundamental aspects of neuroautonomic regulation and sympatho-vagal balance common for mammalian species.

### Conclusion

We use the DFA method to understand activity patterns in mice during waking and resting SWS state. Both states are marked by a high correlation (HC) pattern for short time scale and subsequent anti-correlated (AC) behaviour at large time scale. During wake we credit the HC pattern to the coordinated skeletal body movement. Exclusively for waking state, we detect a multifractal behaviour which we attribute to the composition of stereotyped movements with distinct characteristic scales. The AC regime during waking state is probably related to physiologic behavioural strategy of alternating periods of predominant movement or rest. During SWS, the DFA curve begins with HC, which is mainly attributed to cardiac and respiratory coordinated movements. For both wake and SWS the observed HC regime, which encompasses at least two decades at time scales and is characterized by very different fractal exponents for wake and SWS, is indicative of a critical behaviour with a phase transition that has been reported also in that physiological systems [52], [53].

We believe that our work will be helpful to future studies of behavioural refinement of the ethogram or concerning the action of drugs over muscle activity and motor behaviour. Once we have a clear benchmark of the normal DFA curve of mice in waking and SWS state we can analyse the effect of drugs on motor activity. Good examples of motor diseases which could be studied in the light of DFA analysis in animal models could include Parkinson, Huntington chorea, Alzheimer and tardive dyskinesia.

## Materials and Methods

### Animals

In these series of experiments there were used adult male 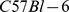 mice (2–5months). Animals were housed in home cages after surgery in 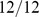 hs light/dark schedule, lights on at 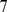 a.m. and food and drink ad libitum. This study was carried out in strict accordance with the recommendations in the Guide for the Care and Use of Laboratory Animals of the National Institutes of Health. The protocol was approved by the Committee on the Ethics of Animal Experiments IINN Intenational Institut of Neuroscience of Natal- Edmond and Lily Safra (Permit Number: 08/2010). All surgery was performed under isoflurane anaesthesia, and all efforts were made to minimize suffering.

### Behavioural recordings, synchronization and identification of wake-sleep states

Behaviours were recorded using a Panasonic videocamera and AMcap software in behavioural groups. In surgery groups, video recordings were synchronized to Local Field Potentials (LFPs) and accelerometer data. This three parameters were used together for confirmation of sleep-wake states selection given by LFPs waves. Spectral analysis of sleep-wake cycle was used to identify and quantify occurrence of waking, paradoxical sleep or REM and slow wave-sleep (SWS) states, using Plexon system for multiple LFP channel processing. Online LFP spectral maps for the characterization of waking and REM/SWS sleep states in mice were employed [54]. Animal behaviour and LFP were continuously observed and recorded in real time for 12 hours. The first 4 recording hours was used in this study for comparisons among treatments. Animals were grouped according to the following categories: WK (active exploration of the environment perceived by video and accelerometer with whisking and hippocampal alpha/theta rhythm), SWS (stillness with eyes closed and large-amplitude slow hippocampal oscillations) and REM sleep. To find the resting SWS sleep intervals we use short time windows in which motor activity remained constant. That means, we use accelerometer record to choose time windows that do not show arousals.

### Open field apparatus

After one week of surgery, animals were anaesthetized with isoflurane, for connecting the tungsten wires of the electrode matrix with those of plexon multielectrode recording device. After that, animals were injected with saline (NaCl 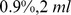) and were placed in an open field apparatus (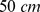 diameter and 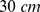 high) at 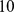 a. m. and recorded for 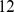 hours. This injection was made because a saline-injected animal can serve as a golden parameter for comparison with drug injected ones in further studies. During this time, animals were not disturbed in order to allow them to disclose in a free manner all the typical movement patterns of SWS and REM sleep, or waking state.

### Multielectrode implantation surgery

Animals were implanted with 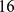 chronic electrodes in the hippocampus (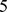 electrodes), motor and somatosensory cortex (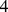 electrodes each one) for intracranial local field potentials (LFPs) recordings. Matrix dimensions were 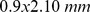, and electrodes were of 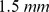 length, composed of 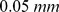 diameter tungsten coated wires, that were attached to an omnetics 16-pin connector. This matrix was implanted in a rectangular hole in the skull (bregma coordinates 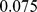 and 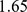 right, and 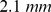 caudal, thus repeating the first coordinate points), under isoflurane anaesthesia. An electrode matrix was placed in animals head using liquid acrylic also with the help of three screws that were used together as ground. Also, an accelerometer with X,Y, Z axes orientation sensor was associated to the headstage, attached to male connector plugged in a head female omnetics connector. Complementarily, a ten fold pre-amplification circuitry was located in association 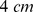 distant from animals head, in order to reduce noise. The LFP signal at a sample rate was amplificated in a second time by a Plexon 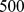 x pre-amplifier and recorded in a 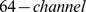 Plexon system for neural recording analysis.

### The accelerometer device

We use a three axis accelerometer sensor (ADXL330 from Analog Devices) installed over to the connector that matches over the mice implanted electrode matrix. This provides a very tight mechanical connection to the animal head, for activities measurement. The three signals were routed from the accelerometer to the headstage using appropriated flexible wires to keep a space about 30 mm. The headstage is home-made and was designed to have 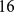 channels by the use of high input impedance operational amps, and its output was electrically compatible to the plexon electrophysiological measurement system. This provides simultaneous recordings for electrophysiological signals and activities. During experiments, the headstage and its cable were kept suspended by the use of a rubber band. A correct adjusts for its strength can avoid a substantial mass load to the head of the animal. This configuration provided a good degree of flexibility and comfort for animal movements during the experiments. Both electrophysiological and inertial signals were initially conditioned in the headstage and then routed to the plexon electrophysiological system. The three axis accelerometer signals from the sensor were low pass filtered to have a 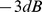 frequency limit around 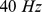, and for convenience all signals were acquired at a rate of 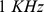.

### Histological preparations

After all behavioural data collection, all surgery animals were subject to histological preparations. Briefly, after electrophysiology recordings, animals were euthanatized in deep anaesthesia with isoflurane, and perfused with PBS pH 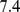, and paraformaldehyde 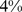; brains were removed, freezed at 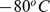 and sliced at 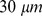 thickness. In order to confirm if electrodes were localized near CA1 hippocampus layers or at 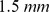 deep in the cortex Nissl staining of coronal slices was performed.

### Data acquisition and treatment

This paper works with measurements of accelerometer sensor located at the head of a mouse and we use accelerometer records to make inference about physiologic state of the organism. A naive, but essential question, is what an accelerometer record? The answer of this question is not movement. The acceleration is the variation of velocity, a particle that moves at constant velocity shows zero acceleration. Acceleration, in a crude way, is related with force. In order to vary the velocity of a particle, or the limb, head or tail of an animal, it is necessary to use muscular force. Besides, an animal can stay at the same place in space but if it shakes or trembles the head the accelerometer shows intense response. In a physiologic perspective the accelerometer records skeletal muscular activity of the body. In other words, the accelerometer is a noninvasive apparatus that quantify motor activity of an organism. The accelerometer output consists in three components of the acceleration vector 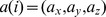. For starting our mathematical analysis of data we combine the vector components in a single quantity similar to the vector module at the same time that we extract the average of each vector component. The new 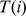 vector formed from the components of 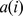 is defined as:
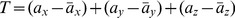
(1)where 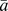 is the arithmetic average of 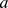. We performed DFA as well Fourier analysis over each of the components of the acceleration and over 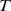 the results are similar, our option for using 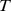 comes from the robustness of the results, an analysis over the modulus of a vector is more trustful than over any of its components.

### Mathematical analysis

*The Detrended Fluctuation Analysis DFA* is an improvement of the rescaled range method used to compute the Hurst exponent. The DFA estimates Hurst exponent in a more rigorous way because it eliminate the trend in the signal before computing deviations if the signal relative to its average [55]–[58]. In what follows we expose DFA in some detail, let start with the a time series 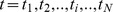 for 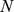 the length of the vector. The first step consists in integrating the series:
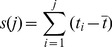
(2)


Where 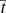 is the local average. Furthermore the new time series is divided into equal boxes of size 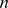. Inside each box of length 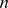 a least-squares line is fitted using a linear function, 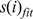 which is called the local trend. The DFA can be adapted to detrend any continuous function, due to the bounded characteristic of the accelerometer signal a linear detrend is enough. We detrend 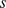 by subtracting it from the local trend, that means:
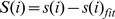
(3)


For any box size 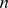 we estimate the root mean square fluctuation
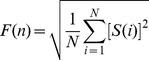
(4)


The equation above is computed for any box of size 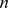; at the end we have a scaling relation 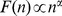, where 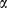 is the DFA scaling exponent and this is a identical to the Hurst index [18], [59], which is related to the power spectrum exponent 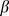 by 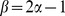 [60], [61]. Furthermore, the exponent 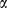 represents the scaling of fluctuation 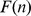 according to the scale 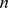. The interpretation of 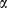 in the statistical mechanic context is: 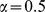 corresponds to the uncorrelated white noise, the result we should expect in a null model perspective or the case of complete randomness. The case 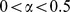 corresponds to anti-correlation while 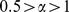 shows positive correlation. The special case 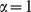 corresponds to 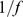 noise [62].

To estimate the multifractal properties of our experimental data we have calculated the multifractal spectra based on the *Multifractal Fluctuation Analysis MF-DFA* method [33], [36], [63]. In a brief description of the MF-DFA method for a given time 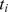 contain N data points as follows. First we integrate the time series 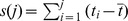, 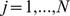. Then we divide the new time series 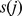 into a non-overlapping segments of size 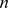, so that 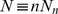. After we apply a linear regression for 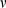-th segment (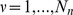) to estimate the best fitting 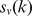, where 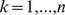. In the next step we calculate the mean square fluctuation for the 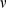-th segment as:
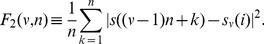
(5)


The last step we calculate the q-th order fluctuation function as:
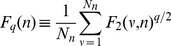
(6)


The scaling of the fluctuation function as we have shown above but now for the moment q is given by:
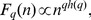
(7)where 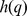 represents a generalized Hurst exponent. Monofractal time series have a unique Hurst exponent 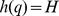 and this is a identical to the DFA scaling exponent 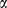. In turn, for multifractal time series the value of 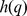 depends nonlinearly on q. From this point, the multifractal scaling exponent 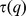 can be calculate from 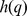 by the relation:
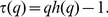
(8)


So, if there is a linear dependence of the spectrum 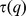 with q, the time series is considered monofractal, otherwise it is multifractal. Moreover, it is possible to characterize the multifractality by considering the multifractal spectrum 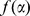, where 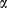 is the Holder exponent. The multifractal spectrum 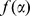 can be obtained from a Legendre transform of the 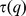 exponent:
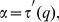
(9)

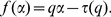
(10)


The magnitude of the multifractality in time series is usually estimated by the width of the spectrum 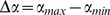 [64]. We deal in this paper with measurement of accelerometer record with an acquisition rate of 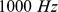. The typical time (period) corresponding to this frequency is 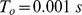. To compute 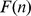, for 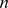 the time scale, we start with 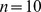 which corresponds to a time 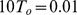, which is the minimal time depicted in the figures of the manuscript. Indeed, to construct 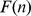 it is necessary to make a statistical average and 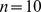 is a minimal statistical sample that produces trustable results.
